# Computerized Dual-Task Testing of Gait Visuomotor and Cognitive Functions in Parkinson’s Disease: Test-Retest Reliability and Validity

**DOI:** 10.3389/fnhum.2021.706230

**Published:** 2021-07-15

**Authors:** Mayank Bhatt, Bhuvan Mahana, Ji Hyun Ko, Tiffany A. Kolesar, Anuprita Kanitkar, Tony Szturm

**Affiliations:** ^1^College of Rehabilitation Sciences, University of Manitoba, Winnipeg, MB, Canada; ^2^Department of Human Anatomy and Cell Science, University of Manitoba, Winnipeg, MB, Canada

**Keywords:** treadmill walking, spatiotemporal gait variables, cognitive performance, dual-task performance, Parkinson’s disease, intra-class correlation coefficient

## Abstract

**Background:**

Mobility and cognitive impairments in Parkinson’s disease (PD) often coexist and are prognostic of adverse health events. Consequently, assessment and training that simultaneously address both gait function and cognition are important to consider in rehabilitation and promotion of healthy aging. For this purpose, a computer game-based rehabilitation treadmill platform (GRP) was developed for dual-task (DT) assessment and training.

**Objective:**

The first objective was to establish the test-retest reliability of the GRP assessment protocol for DT gait, visuomotor and executive cognitive function in PD patients. The second objective was to examine the effect of task condition [single task (ST) vs. DT] and disease severity (stage 2 vs. stage 3) on gait, visuomotor and cognitive function.

**Methods:**

Thirty individuals aged 55 to 70 years, diagnosed with PD; 15 each at Hoehn and Yahr scale stage 2 (PD-2) and 3 (PD-3) performed a series of computerized visuomotor and cognitive game tasks while sitting (ST) and during treadmill walking (DT). A treadmill instrumented with a pressure mat was used to record center of foot pressure and compute the average and coefficient of variation (COV) of step time, step length, and drift during 1-min, speed-controlled intervals. Visuomotor and cognitive game performance measures were quantified using custom software. Testing was conducted on two occasions, 1 week apart.

**Results:**

With few exceptions, the assessment protocol showed moderate to high intraclass correlation coefficient (ICC) values under both ST and DT conditions for the spatio-temporal gait measures (average and COV), as well as the visuomotor tracking and cognitive game performance measures. A significant decline in gait, visuomotor, and cognitive game performance measures was observed during DT compared to ST conditions, and in the PD-3 compared to PD-2 groups.

**Conclusion:**

The high to moderate ICC values along with the lack of systematic errors in the measures indicate that this tool has the ability to repeatedly record reliable DT interference (DTI) effects over time. The use of interactive digital media provides a flexible method to produce and evaluate DTI for a wide range of executive cognitive activities. This also proves to be a sensitive tool for tracking disease progression.

**Clinical Trial Registration:**

www.ClinicalTrials.gov, identifier NCT03232996.

## Introduction

Safe, independent community walking requires both mobility skills and cognitive flexibility to manage complex terrains, for navigation in busy environments, to identify and track relevant visual targets, reading, and for processing of what is being seen. Mobility limitations (balance and gait impairments; Rochester et al., 2004; Kang et al., 2005) and decline in executive cognitive function (Olchik et al., 2017; Roheger et al., 2018) common to Parkinson’s disease (PD) often coexist (Plotnik et al., 2011; Schneider et al., 2015) and are prognostic of adverse health events, including falls (Mak et al., 2014; Paul et al., 2014). Over 60% of individuals with PD fall each year and a significant portion of those who fall will experience multiple falls (Pickering et al., 2007; Lord et al., 2017; Creaby and Cole, 2018). The consequences of falls are often severe, leading to disability, loss of independence, and social isolation.

The concept of motor automaticity has an important implication in the pathophysiology of PD related to mobility limitations and increased fall risk (Wu et al., 2015; Gilat et al., 2017). One model of executive cognitive functions presents the supervisory attentional system, which distinguishes between processing of non-routine, attentionally demanding activities vs. routine, automated tasks (Dirnberger and Jahanshahi, 2013). The role of the striatum has been equated with that of routine tasks, such as locomotion, that are usually performed automatically (Gilat et al., 2017). The information processing demands of community ambulation are presumed to affect the already limited locomotor control of PD patients (Rochester et al., 2004; Kang et al., 2005), and this may result in greater gait instability and a higher risk of falls (Heinzel et al., 2016; Penko et al., 2018). Consequently, dual-task (DT) assessment and training programs that address both mobility and cognition are important to consider in the prevention and rehabilitation of mobility and cognitive decline.

Impairments in gait (Rosenberg-Katz et al., 2015; Wu et al., 2015; Gilat et al., 2017), and in executive functions (Monchi et al., 2001, 2004) are associated with disruption of frontostriatal circuits in PD. This is consistent with findings of studies which have evaluated frontal lobe activity during DT-walking using functional near infrared spectroscopy (fNIRS). Several fNIRS studies have reported increased activation levels of the prefrontal cortex (PFC) during DT-walking as compared to walk-only trials in healthy able-bodied adults (Fraser et al., 2016; Maidan et al., 2016). Similar fNIRS studies evaluating PD patients have reported no difference in PFC activation levels for DT-walking trials as compared to walk-only trials (Holtzer et al., 2015; Maidan et al., 2016; Liu et al., 2021). A recent positron emission tomography pilot study of DT-walking evaluated brain glucose metabolism in PD (Hoehn and Yahr stages 2 and 3) in ten patients who rested and five who conducted a DT treadmill/cognitive video game task during the uptake period (Szturm et al., 2020). DT interference (DTI) was consistently shown for gait and cognitive game performance measures for the DT-walking group. As expected, glucose metabolism was significantly increased in several brain regions of the patients in the DT group, compared to the resting group, including the primary visual/sensorimotor areas, thalamus, superior colliculus, and cerebellum. Within the DT group, the three patients in the earlier stage of PD (Hoehn and Yahr stage 2) showed increased glucose uptake in the PFC during DT treadmill walking compared to the resting group, while the two patients at Hoehn and Yahr stage 3 did not. These behavioral and neuroimaging results suggest that simultaneous tasks may be accomplished via different mechanisms as neurodegeneration progresses in PD. A clinically valid and reliable treatment to assess and improve DT performance may translate to improved or slowed decline of cognitive performance and mobility.

Most DT gait studies are performed overground and all overground studies report a significant decrease in gait speed during the DT-walking condition as compared to walk only trials (Al-Yahya et al., 2011; Rochester et al., 2014; Stegemöller et al., 2014; Salazar et al., 2017; Raffegeau et al., 2019). Besides gait speed, many DT gait studies examine how cognitive demand affects gait rhythm and stability (i.e., recording spatiotemporal gait variables; Frenkel-Toledo et al., 2005; Montero-Odasso et al., 2012; Nankar et al., 2017). However, gait speed is a confounding variable as spatiotemporal and other kinematic gait variables are sensitive to changes in gait speed (Jordan et al., 2007; Chung and Wang, 2010; Keene et al., 2016; Cole et al., 2017). Most of the overground DT-walking studies use an instrumented walkway, which records only 4–6 consecutive steps (Bruijn et al., 2009; Hollman et al., 2010). It has been shown that using a continuous walking protocol instead of short intermittent walks, and collecting more than 30 consecutive steps improved reliability, in particular for measures of gait variability (Bruijn et al., 2009; Hollman et al., 2010; Galna et al., 2013). Furthermore, there is a limited choice of executive cognitive tasks that can be completed during overground walking, and for the short time period to walk a few meters. For example, common cognitive tasks used during overground walking include recall of names/words, serial subtraction, or auditory Stroop tests (Al-Yahya et al., 2011; Raffegeau et al., 2019). Walking outdoors requires visual attention, search, and processing of what is being seen (i.e., relevant visual information). Cortical areas devoted to visuospatial processing of behaviorally significant information, such as the PFC may be particularly susceptible to the neurodegenerative process in PD (Klencklen et al., 2012; Battisto et al., 2018; Lally et al., 2020). To overcome these limitations, Szturm et al. (2017, 2020) developed a computer game-based rehabilitation treadmill platform (GRP; Mahana et al., 2021) which provides an integrated approach to assess and treat the decline in balance, gait, visuomotor, and executive cognitive functions. The GRP consists of a standard treadmill instrumented with a pressure mapping system used to compute spatiotemporal gait variables and gait stability measures (Szturm et al., 2017; Ahmadi et al., 2018, 2019) and a computer display placed at the front of the treadmill for participants to view and interact with computer-generated activities while walking. The GRP includes an interactive computer game application for DT which allows for recording and analyzing participant’s actions and choices while walking and playing targeted cognitive games. Therefore, gait and cognitive performance can be measured synchronously during continuous, speed-controlled, steady state gait trials, lasting minutes.

In a previous study (Szturm et al., 2017), reliability of the GRP assessment tool has been established in older adults with fall histories. The present study aims to establish the reliability of the GRP assessment protocol in individuals with PD, and to examine the influence that visuospatial cognitive tasks have on gait performance in Hoehn and Yahr stage 2 (PD-2) vs. stage 3 (PD-3) patients. The first objective of the present study was to establish test-retest reliability and the minimal detectable change (MDC) of spatio-temporal gait variables, as well as visuomotor and cognitive performance measures examined during single task (ST; i.e., walking only or playing video games while sitting) and DT (i.e., walking while playing video games) conditions. The second objective was to examine the effects of task condition (ST vs. DT conditions) and disease severity (stage 2 vs. 3) on gait, visuomotor and cognitive performance measures. This objective addressed three hypotheses, which are as follows: (1) increased visuospatial cognitive loads (ST-walking vs. DT-walking) will have a significant effect on gait performance measures in both PD-2 and PD-3 patients, and *vice versa*: increased physical demands (sitting vs. walking) will have a significant effect on visuomotor and cognitive performance measures (ST-gaming vs. DT-gaming); (2) increased cognitive loads (ST-walking vs. DT-walking) have a greater influence in gait performance measures in PD-3 patients as compared to PD-2 patients; and (3) increased physical demands (sitting vs. walking) will have a greater influence on visuomotor and cognitive performance measures in PD-3 patients as compared to PD-2 patients.

## Materials and Methods

### Participants

Thirty PD patients were recruited for this study. The inclusion criteria included: (a) 50–75 years of age, (b) clinical diagnosis of PD (Hoehn and Yahr stages 2 and 3) made by a movement disorders specialist based upon the United Kingdom PD Society Brain Bank diagnostic criteria (Hughes et al., 1992), (c) treated with antiparkinsonian medications, (d) able to walk at least 50 m without any assistance, and (e) adequate hearing and vision to perform the computer game task, i.e., while viewing an 80 cm monitor at a distance of 1 m. The exclusion criterions included (a) any significant cognitive impairment [Montreal cognitive assessment scores (MoCA; Nasreddine et al., 2005) <25], (b) any other neurological disorder except PD, (c) with any musculoskeletal impairment or uncontrolled cardio-vascular condition that might prevent participants from walking on a treadmill for 2–4 min. The Human Research Ethics Board at the University of Manitoba approved the study, and the study is registered at ClinicalTrials.gov. The Protocol Registration System number is NCT03232996. All participants provided informed consent. The participants were tested on two separate days, 1 week apart, and at the same time of day, during the ON medication phase. The Unified Parkinson’s Disease Rating Scale (UPDRS; Fahn et al., 1987) was completed in the first test session. Participants were instructed to take their medications at the same time prior to testing.

### Instrumentation and Test Protocol

**FIGURE 1:**
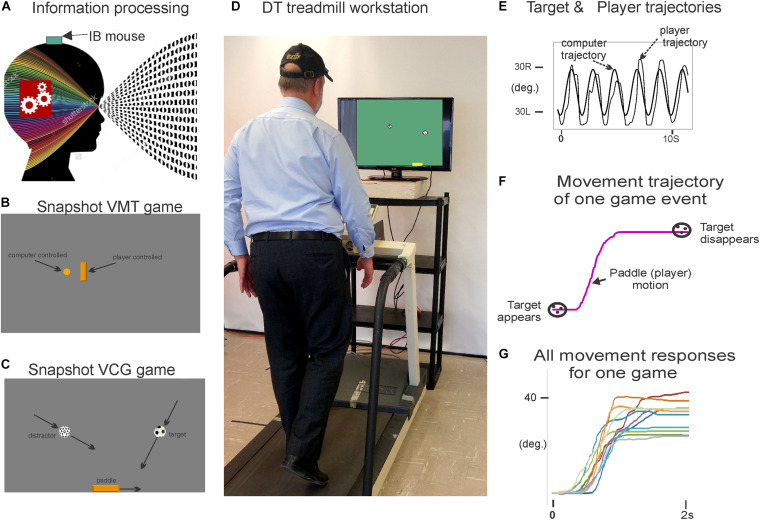
Illustration of the treadmill platform and experimental set-up; **(A)** the motion mouse is shown attached to the head via a baseball cap. **(D)** Participant is walking on the instrumented treadmill while viewing a computer monitor. Head rotation (via motion mouse) is used to interact with the visuospatial cognitive task. **(B,C)** present snap shots of the VMT and VCG. **(E)** Presents synchronous plots of the target cursor motion and user movement trajectories (head rotation) for a typical VMT task. Maxima are the left most position and minima the right most position. **(F)** Presents the trajectory for one game movement response from target appearance to target disappearance. **(G)** Presents overlay trajectories of all game movement responses for one game session. Parts of Figure 1 have previously been published in Szturm et al. (2017, 2021).

Figure 1 presents the components of the treadmill platform. Participants stood on a treadmill at a viewing distance of 100 cm from an 80 cm computer monitor. The treadmill is instrumented with a pressure mat (Vista Medical, Ca) which was used to record vertical foot contact forces and to compute spatiotemporal gait variables (Rochester et al., 2014). A miniature, wireless inertial-based (IB) mouse Scoop Pointer Remote (Model: RXR1000-0302E; Hillcrest labs) was used to control and interact with two computer applications described below. The IB mouse contains a 2-axis gyroscope and 2-axis accelerometer and firmware that provides an instantaneous angular position signal, and emulates a plug-n-play standard optical computer mouse. Velcro was used to secure the wireless motion mouse to a sports cap or plastic head band allowing head rotation to be used to point the device to control the position and motion of a computer cursor or game sprite. Therefore, a hands-free computer game controller was used to interact with various computer applications (cognitive activities) while treadmill walking. Head pointing movements are among the most natural and can easily be performed with minimal instruction.

Two computer applications were developed for DT balance assessment. Details of the applications and protocols can be found in Szturm et al. (2017). First, visuomotor tracking (VMT), as shown in Figures 1B,E, involves tracking a visual target that moves horizontally left and right on a computer display for several cycles. Two cursors of different shapes appear on the monitor. The target was a circle, its motion was computer controlled and moved at a predetermined frequency of 0.5 Hz with an amplitude of 80% of the monitor width. The second cursor, a rectangle, was slaved to head rotation via the IB mouse. Participants were instructed to move and overlap the rectangle head-controlled cursor with the target circle for several cycles. The second computer application involved a visuomotor cognitive game (VCG), as shown in Figures 1C,F,G. The goal of this task is to move a game paddle (via head rotation) horizontally to interact with the moving game objects. The game objects are categorized as designated targets or designated distractors. The game objects appear at random locations at the top of the display every 2 s and move to the bottom of the display. The game objects move in a diagonal path from top to bottom. In response to each game event, participants produced a head rotation (i.e., rotation of the IB mouse) to move the game paddle to catch the target objects while avoiding the distractors. A logged data file is generated that synchronously records (100 Hz sampling rate) the coordinates of the game paddle and the game objects (tracking cursor, target, and distractor objects).

These tasks were performed while walking on a treadmill at 0.9 m/s for 1 min. This speed was well tolerated by the PD-3 group in pilot-testing, allowing these patients to walk without falling or needing to lean on the treadmill supports. Four conditions were included: (a) walk only ST condition (WO), (b) DT-walk while performing a VMT task, (c) DT-walk while performing a VCG task, and (d) a ST gaming condition while seated (VMT and VCG games). Participants were not allowed to lean on the treadmill structure during testing. Prior to testing, the participants were allowed to play the games while seated for a few minutes to become familiar with each task.

### Data Analysis

The following outcome measures were computed from the recorded pressure mat data; (a) average and coefficient of variation (COV) were computed over 30 consecutive steps, for right and left step length (SL) and swing time (SwT; 44), and (b) the location of all heel contacts in the mediolateral (ML) axis during each walk trial were determined, and the dispersion of heel contact locations were computed as the COV, and reported as ML-drift (Ahmadi et al., 2018). Since the statistical analysis showed no significant difference in means of right and left gait variables, only the right gait variables are presented.

#### VMT Performance Measures

Figure 1E presents synchronous plots of the computer-controlled target motion (circle cursor) and participants head rotation (rectangle cursor) for a typical VMT task. The total residual error (TRE) was determined by computing the difference between the trajectories of the target and head cursor motions for each sample period (Szturm et al., 2017). The first two cycles of the VMT tasks were excluded to allow the participants time to acquire the moving target and begin tracking.

#### VCG Performance Measures

Figure 1F presents the trajectory of an individual game movement response. The duration of each VCG trial was 60 s and each game event was 2 s. Therefore, 30 game movement responses were made, half in each direction. Based on the time indexes of each target appearance and disappearance, the software segments each individual game movement response and sorts them by direction. Figure 1G presents overlay plots of all game movement responses in one direction (Szturm et al., 2017). The following variables were determined from the game movement responses: (a) average response time (Avg. RT), i.e., the time from target appearance to the start of the game paddle (head rotation), (b) success rate (SR) determined as the percentage of target objects that were caught, and (c) movement variance (MV). The individual movement traces for each direction in one game session were averaged and the standard deviation (SD) computed for each sampled data point. The total SD over all sampled data points was taken as movement variation.

### Statistical Analysis

The sample size for this study was computed using Table 1, of Zou (2012). Thirty participants were required for an intraclass correlation coefficient (ICC) value of 0.8, assurance of 70%, and class interval half-width of 0.15. Normal distribution of the data was assessed using the Shapiro–Wilk test, and Mauchly’s test of sphericity was used to test the equality of variance for comparison between different task conditions and stages of PD. The gait, VMT and VCG performance measures satisfied the assumptions of normality and equal variance.

**TABLE 1:** Demographic and clinical data by Hoehn and Yahr stage (stage 2: PD-2; stage 3: PD-3).

	**PD-2**	**PD-3**	***p-value* (PD-2 vs. PD-3)**
Total participants	15	15	
Age (mean ± SD) years	63.2 ± 5.5	66.6 ± 5.6	0.105
Male/Female	9/6	10/5	0.705
Time since diagnosis (years)	4.4 ± 4.1	6.7 ± 4.4	0.150
UPDRS Total (mean ± SD)	28.6 ± 9.9	43.6 ± 9.6	<0.001
UPDRS Motor (mean ± SD)	20.9 ± 7.7	30.8 ± 6.6	<0.001
MoCA (mean ± SD)	29.0 ± 1.0	28.7 ± 1.0	0.418

Relative reliability was assessed using a two-way random model ICC. The ICC scores were considered to be high when equal to or greater than 0.70, moderate between 0.50 and 0.69, and low when less than 0.50 (Lexell and Downham, 2005). MDC was computed according to a published guideline (Lexell and Downham, 2005). Absolute reliability was analyzed using MDC (Messick, 1995; Haley and Fragala-Pinkham, 2006). Systematic errors between the test periods were evaluated using a paired *t*-test.

A repeated measures ANOVA was used to evaluate the effect of task condition (ST vs. DT), disease severity (Hoehn and Yahr stage 2 vs. 3) and the interaction of task and disease stage. The significance level was α set at *p* < 0.05. Effect size was calculated (Cohen’s *d*); values less than 0.20 were considered negligible, values between 0.20 and 0.50 were considered small, between 0.50 and 0.80 were medium, and greater than 0.80 was considered large (Schäfer and Schwarz, 2019). All statistical analysis was conducted using SPSS (v.22, SPSS Science, Chicago, IL, United States, RRID:SCR_019096).

## Results

Table 1 presents the demographic and clinical data for PD-2 and PD-3 participants. The male to female ratios were similar in the two PD groups. There was a slight age difference between the two groups but this was not statistically different [*t*(28) = 1.678, *p* = 0.105]. Test scores for the MoCA were similar between groups (*p* > 0.7). The UPDRS scores (Total and Motor) were significantly greater in the PD-3 group as compared to the PD-2 group (*p* < 0.001).

Table 2 presents group means and SD for tests 1 and 2, ICC values, and MDC values for gait variables (average and COV for SL and SwT and ML-drift). High test-retest reliability (ICC values of 0.76–0.98) were observed for average SL and SwT during the three-walking conditions: ST-walking only (WO), VMT-walking and VCG-walking. MDC as a percentage of the group mean (MDC%) ranged from 15–20% for Avg-SL and Avg-SwT. Moderate to high test-retest reliability (ICC values of 0.58–0.96) was observed for COV-SL and COV-SwT during the three walking conditions. The MDC% values for COV were higher than for average gait values; values ranged from 52–74%. Lower ICC values were observed for the ML-drift measures; range 0.43–0.88. The MDC% values for ML-drift were high, ranging 67–75%. Paired student *t*-tests revealed no significant difference between test 1 and test 2 for any of the gait variables (average, COV, or ML-drift, *p* > 0.2).

**TABLE 2:** Test-retest reliability results for spatial and temporal gait outcome measures (average and COV).

**Condition**	**Test 1 mean (SD)**	**Test 2 mean (SD)**	**ICC Value (95% CI)**	**MDC (MDC%)**
**Average SL (cm)**				
WO	45.9 (13.6)	44.7 (14.1)	0.92 (0.84–0.96)	9.1 (20)
VCG	43.3 (16.8)	41.6 (16.4)	0.97 (0.93–0.98)	7.9 (18)
VMT	42.1 (13.8)	46.3 (14.7)	0.91 (0.81–0.96)	8.3 (19)
**Average SwT (s)**				
WO	0.72 (0.1)	0.70 (0.1)	0.91 (0.81–0.96)	0.12 (16.7)
VCG	0.65 (0.1)	0.65 (0.1)	0.90 (0.76–0.95)	0.10 (15.4)
VMT	0.66 (0.1)	0.67 (0.1)	0.90 (0.76–0.95)	0.11 (16.7)
**COV SL (%)**				
WO	26.2 (14.3)	24.6 (13.1)	0.81 (0.58–0.91)	18.2 (70)
VCG	36.5 (21.5)	35.8 (21.1)	0.90 (0.77–0.95)	18.8 (52)
VMT	36.1 (16.5)	34.0 (14.4)	0.87 (0.71–0.94)	23.1 (64)
**COV SwT (%)**				
WO	10.9 (6.1)	11.1 (5.6)	0.89 (0.76–0.95)	7.5 (69)
VCG	12.5 (6.7)	12.7 (7.3)	0.84 (0.64–0.92)	8.8 (70)
VMT	13.1 (9.1)	13.1 (9.1)	0.91 (0.80–0.96)	9.5 (74)
**ML-Drift (%)**				
WO	13.9 (7.7)	13.4 (6.8)	0.63 (0.43–0.83)	9.2 (67)
VCG	18.6 (9.4)	17.1 (10.4)	0.74 (0.55–0.88)	14.1 (75)
VMT	16.3 (7.5)	17.7 (8.1)	0.67 (0.45–0.85)	11.8 (73)

*Presented are the group mean (standard deviation, SD) for test 1 and test 2 (occurring 1 week later), intraclass correlation coefficient (ICC) values (95% confidence interval, CI), and minimal detectable change (MDC% as a percentage of the group mean). The values are for single-task walking only (WO), dual-task visuomotor cognitive game (VCG), and dual-task visuomotor tracking (VMT) tasks.*

Table 3 presents the ICC values and MDC values, along with group means and SD for both the VMT (TRE) and VCG (movement onset time, success rate, and movement variation) outcome measures. Moderate to high test-retest reliability (ICC values ranging from 0.48 to 0.95) were observed for TRE. The MDC% values for TRE were 19% for sitting and 21% for VMT-walking. Moderate to high test-retest reliability was observed (ICC values ranging from 0.41 to 0.95) for movement onset time, success rate, and movement variation. The MDC% values for movement onset time and success rate ranged from 11 to 20%, and for movement variation MDC% was 20% in sitting and 19% during walking. Paired student *t*-tests revealed no significant differences between test 1 and test 2 for any of the VMT or VCG outcome measures (*p* > 0.3).

**TABLE 3:** Test-retest reliability for visuomotor tracking (VMT) and visuomotor cognitive game (VCG) task performance during ST-sitting and DT-walking on the treadmill.

**Condition**	**Test 1 mean (SD)**	**Test 2 mean (SD)**	**ICC Value (95% CI)**	**MDC (MDC%)**
**Visuomotor tracking performance**				
**Total residual error**				
Sitting	11.3 (2.2)	10.9 (2.6)	0.88 (0.74–0.95)	2.2 (19)
Walking	14.6 (3.2)	13.3 (3.1)	0.76 (0.48–0.89)	3.1 (21)
**Visuomotor cognitive game performance**				
**Movement onset time**				
Sitting	0.6 (0.1)	0.6 (0.1)	0.85 (0.68–0.93)	0.07 (11)
Walking	0.7 (0.1)	0.6 (0.1)	0.55 (0.41–0.71)	0.14 (20)
**Success rate**				
Sitting	82.9 (15.4)	86.7 (14.2)	0.85 (0.65–0.93)	16.5 (20)
Walking	73.4 (15.9)	72.5 (19.2)	0.89 (0.77–0.95)	14.4 (19)
**Movement variation**				
Sitting	18.1 (2.2)	18.8 (3.1)	0.60 (0.45–0.72)	3.7 (20)
Walking	21.9 (3.8)	22.1 (3.5)	0.85 (0.67–0.93)	4.1 (19)

*This table demonstrates the mean (standard deviation, SD) for test 1 and test 2, intraclass correlation coefficient (ICC) values (95% confidence interval, CI), and minimal detectable change (MDC% as a percentage of the group mean).*

### Validity

Statistical results of the effect of task condition (WO vs. VMT/VCG), disease stage (PD-2 vs. PD-3), and task × stage interaction on the gait variables are presented in Table 4. There was a significant main effect of task condition on all gait performance measures (*p* < 0.05) except for ML-drift between WO and VMT conditions (*p* = 0.1). There was a significant main effect of disease stage on all gait outcome measures (*p* < 0.05) except for Avg-SwT (*p* = 0.1). With one exception (COV of SwT for the VCG task, *p* = 0.008), there was no interaction effect (task × stage) on any of the gait outcome measures (*p* > 0.1).

**TABLE 4:** Main effects and effect sizes (Cohen’s *d*) of task condition and PD severity on the gait outcome measures.

**Gait outcome measures**	**Task condition**			**PD stage**			**Interaction (Task × Stage)**		
	***F***	***p***	***d***	***F***	***p***	***d***	***F***	***p***	***d***
**Visuomotor tracking performance**									
Avg. SL (cm)	3.8	0.05	0.20	20.5	0.001	0.52	0.3	0.6	0.01
Avg. SwT (s)	8.1	0.01	0.29	0.6	0.5	0.10	0.1	0.8	0.01
COV SL (%)	19.8	0.001	0.51	12.6	0.02	0.34	2.7	0.1	0.11
COV SwT (%)	5.6	0.03	0.25	12.2	0.002	0.36	0.3	0.6	0.12
ML-drift (%)	2.6	0.1	0.19	6.1	0.02	0.27	2.8	0.1	0.16
**Visuomotor cognitive game performance**									
Avg. SL (cm)	2.4	0.1	0.12	19.7	0.001	0.50	1.2	0.3	0.05
Avg. SwT (s)	6.5	0.02	0.26	0.9	0.3	0.04	0.001	0.9	0.01
COV SL (%)	15.7	0.001	0.46	12.6	0.002	0.36	0.1	0.8	0.01
COV SwT (%)	3.7	0.05	0.20	7.4	0.01	0.31	8.4	0.008	0.12
ML-drift (%)	5.0	0.03	0.28	6.5	0.02	0.26	0.5	0.5	0.02

*Avg., average; SL, step length; SwT, swing time; COV, coefficient of variation; and ML-drift, mediolateral drift.*

**FIGURE 2:**
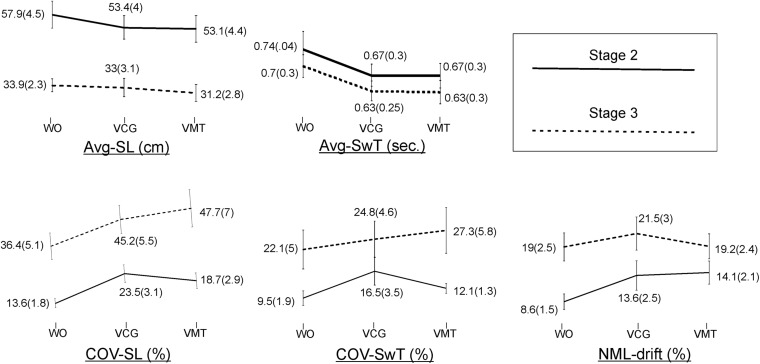
Plots and values of group means and in brackets standard error of mean (SEM) for the gait variables by task condition and disease stage.

Group means and SD of the spatiotemporal gait variables by task condition and disease stage are presented in Figure 2. As compared to the WO condition, the addition of the VMT and VCG tasks resulted in (a) a significant decrease in Avg-SwT and Avg-SL, and (b) a significant increase in COV-SL, COV-SwT, and ML-drift. As compared to the PD-2 group, the PD-3 group had significantly shorter Avg-SwT, larger COV values, and greater ML-drift. This was the case for WO, VMT, and VCG walk trials. Effect sizes for task condition and disease stage were similar, ranging from small to medium (0.20 to 0.52).

The magnitude of DTI (i.e., effects of task condition) of cognitive demands on gait performance measures was similar in both PD-2 and PD-3 groups. The one exception was a greater magnitude of DTI on COV-SwT for the PD-3 group as compared to the PD-2 group. Effect sizes for task × stage interaction were negligible, less than 0.20.

**FIGURE 3:**
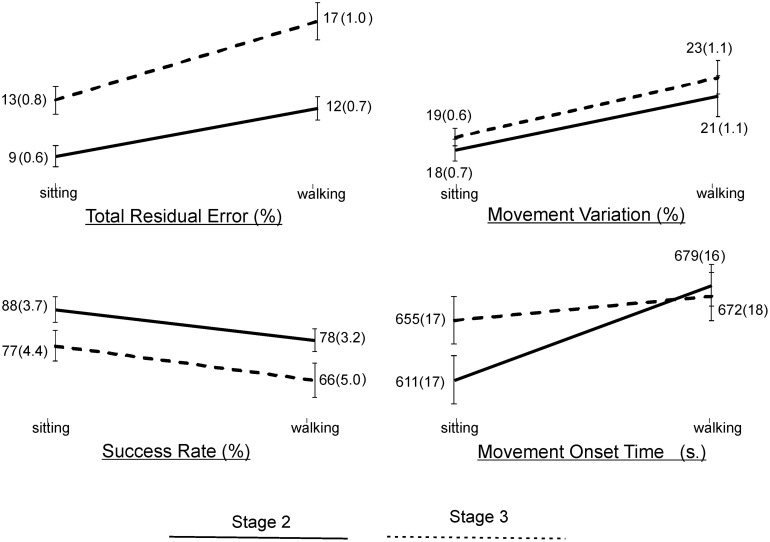
Plots and values of group means and in brackets standard error of mean (SEM) for the VMT and VCG outcome measures by task condition and disease stage.

Statistical results of the effect of task condition and disease stage on VMT and VCG performance measures are presented in Table 5. There was a significant effect due to task condition and disease stage on TRE (*p* < 0.001), but there was no significant task × stage interaction effect for TRE (*p* = 0.2). As evident in Figure 3, TRE was significantly greater during DT-walking as compared to sitting, and was significantly greater in the PD-3 group compared to the PD-2 group. This was the case when tested in sitting and during DT-walking.

**TABLE 5:** Main effects and effect sizes (Cohen’s *d*) of task condition and PD severity on the visuomotor tracking (VMT) and visuomotor cognitive game (VCG) outcome measures.

**Cognitive performance measures**	**Task condition**			**PD stage**			**Interaction (Task × Stage)**		
	***F***	***p***	***d***	***F***	***p***	***d***	***F***	***p***	***d***
**Visuomotor tracking performance**									
Total residual error	26.4	0.001	0.56	16.8	0.001	0.47	1.6	0.2	0.07
**Visuomotor cognitive game performance**									
Movement onset time	8.3	0.009	0.32	0.7	0.41	0.03	2.9	0.1	0.10
Success rate	28.8	0.001	0.58	4.2	0.05	0.20	0.03	0.9	0.01
Movement variation	22.2	0.001	0.52	0.7	0.42	0.03	0.06	0.8	0.01

There was a significant effect due to VCG task condition on movement onset time, success rate, and movement variation (Table 5). There was a significant effect due to disease stage on success rate but not on movement onset time or movement variation. There was no significant task × stage interaction effect on any of the three VCG outcome measures. As evident in Figure 3, there was a significant decrease in success rate and a significant increase in movement onset time and movement variation when tested during DT-walking as compared to sitting. Also, success rate was significantly higher in the PD-2 group compared to the PD-3 group when tested in sitting and during DT-walking. Medium effect sizes for TRE (*d* = 0.56), success rate (*d* = 0.58), and movement variation (0.52) were observed for task condition. Small effects sizes for TRE (0.47) and success rate (0.20) were observed for disease stage. Effect sizes for task × stage interaction were negligible, less than 0.20.

## Discussion

Overall, with a few exceptions, the computer game-based DT treadmill walking assessment protocol showed moderate to high test-retest reliability for the spatio-temporal gait measures, and VMT and VCG performance measures, under the DT condition. DTI was observed for all outcome measures of gait, VMT, and VCG with one exception: ML drift was not significantly different between WO and VMT trials. The limitation of a treadmill is the relatively narrow belt width, which does limit the degree to which a participant can drift leftward and rightward before stepping off the moving belt and falling. This constraint may have influenced the amount of ML drift during the VMT task. However, it is not clear why there was a significant increase in ML drift during the VCG tasks.

The present reliability findings are consistent with the results of Strouwen et al. (2016) who examined test-retest reliability for average SL and average time during overground DT-walking in individuals with PD (Strouwen et al., 2016). Participants walked on a GAITRite instrumented walkway of 4 m while performing backward-digit counting and an auditory Stroop test. Average SL and SwT and the cognitive performance measures (number of errors), demonstrated moderate test-retest reliability during DT-walking. However, it is not known whether gait speed was the same for task conditions and test periods in this study (Strouwen et al., 2016). The present analysis extends these results to include measures of variation: COV-SL, COV-SwT, and ML-drift. In addition, in the present study, participants performed the VMT and VCG tasks for 60 s while walking (DT); the same steady state velocity was used for both DT and ST (walk only) conditions and test periods. Therefore, the present analysis was based on averages and variance of 30 movement cycles (VMT), 30 game movement responses, and on average, 30 consecutive right and left steps. In regard to absolute reliability, MDC% values for the average SL and SwT gait variables for WO, VMT and VCG walking trials were in the range of 15–20%. Values of MDC less than 20% would be considered an acceptable level for use in clinical trials (Koo and Li, 2016). The COV and ML-drift outcome measures had high MDC% values, with a range of 52–75%. When evaluating the effectiveness of treatment programs, outcome measures having high MDC% values would require a greater amount of change from pre- to post-intervention in order to be considered significant. Thus, caution needs to be taken when using gait variability measures to examine change due to some intervention. In this regard, inclusion criteria may need to be restricted (for example to include only Stage 2 or Stage 3 PD patients) as to minimize subject variability.

A significant reduction in average SL, and average SwT was observed when the VMT and VCG tasks were added. This was the case for both PD-2 and PD-3 groups. These findings for average gait variables would indicate a significant effect of visuospatial processing load on locomotor rhythm and pacing. There was also a significant increase in COV of SL, SwT, and ML-drift when the visuospatial processing loads were increased. Gait variability measures are important outcome measures as they reflect gait stability (Frenkel-Toledo et al., 2005; Montero-Odasso et al., 2012, 2018; Ahmadi et al., 2019) and are independent predictors of falls (Callisaya et al., 2011; Heinzel et al., 2016). In addition, previous studies have reported increased ML excursions of the body during treadmill walking when exposed to moving visual scenes (Bauby and Kuo, 2000; O’Connor and Kuo, 2009). Taken together, the present findings for COV-SL/SwT and ML-drift would indicate a main effect of visuospatial processing load on gait stability in PD.

Most studies that examined DT-walking in PD patients have used cognitive tasks such as counting backward tasks, animal/word enumeration tasks, and auditory Stroop tests (Rochester et al., 2014; Stegemöller et al., 2014; Salazar et al., 2017; Raffegeau et al., 2019). The present study extends these tasks to include VMT and VCG tasks, which require participants to actively rotate their heads in order to track (VMT) and to interact (VCG) with moving target objects and avoid distractor objects. Many different types of similar head pointing movements and gaze fixations occur during our daily activities such as walking and navigating through busy and complex environments, avoiding people and obstacles, shopping and searching for specific items, or during many recreational activities. A significant decline in performance of all visuospatial processing tasks was observed when tested during DT-walking, as compared to sitting. This was the case for both PD-2 and PD-3 groups.

Schlachetzki et al. (2017) showed a significant decrease in gait speed, and SL of stage 3 participants as compared to stage 2. Hass et al. (2012) also reported a significant reduction in average SL, and average SwT of stage 3 participants compared to stage 2. The present study extends these findings and demonstrates a significant decline in gait, VMT and VCG performance measures for the PD-3 group compared to the PD-2 group during both ST and DT treadmill walking conditions. However, there was no significant difference in the magnitude of DTI (task × stage interaction) of cognitive demands on gait performance measures between the PD-2 and PD-3 groups. During the WO trials, SL was significantly less and gait variation and ML-drift were significantly greater in the PD-3 group compared to the PD-2 group. This may indicate that the PD-3 group is near the threshold level (capacity) beyond which a fall or loss of balance would occur, i.e., if the magnitude of DTI was higher in PD-3 patients, they would have fallen. For this reason, a greater increase of DTI in the PD-3 group would not be possible.

Following the principle of neural overlap (Wollesen et al., 2016; Crockett et al., 2017), DTI should be greatest when the cognitive and motor tasks engage the same neural circuits and processing resources, e.g., visual-spatial processing. Walking and maintaining balance on a moving treadmill requires attentional resources for continuous monitoring of rhythm, pacing, spatial orientation relative to borders of the treadmill belt, and to generate timely corrective responses to counter drift. This requires processing and organization of spatial information from multiple sensory systems, including visual spatial information. The VMT and VCG tasks used in the present study require sustained visual attention, visual search, cognitive inhibition and spatial processing of the moving targets, i.e., executive cognitive functions. The increased attentional demands and processing of spatial information to prevent drifting and, more seriously, falling, would compete for resources required to perform the VMT and VCG tasks, and *vice versa*.

## Limitations

One limitation of the present study which is true of all DT studies relates to how individuals spontaneously prioritize their attention between the walking and the tracking/cognitive tasks. It is possible that walking was prioritized and participants did not attend to the tracking/cognitive activities with 100% effort. Alternatively, the information provided during the VMT and VCG tasks was received and processed and the performance was affected due to DTI. Modest performance levels were observed in the present study for both VMT and VCG tasks during walking. This would indicate the participants were attending to and processing the information they saw on the display. However, they may have stopped intermittently for a few seconds and prioritized locomotor processing (i.e., to correct rhythm, pacing and balance). Another limitation is that treadmill and overground are different types of walking tasks. However, treadmill walking involves repetitive stepping and dynamic stability requirements that are comparable to the demands of overground walking, as well the kinematics and kinetics of gait during treadmill and overground walking have very similar patterns and degree of variation (Watt et al., 2010; Hollman et al., 2016; Yao et al., 2019). In the present assessment, treadmill was used to ensure a steady state gait at a controlled speed and within a narrow spatial range (width/length of treadmill walking surface). Also important is that treadmills can be easily equipped with a computer monitor to access digital media. This provides standardized repeatable computer tracking and cognitive game activities and thus presents the same information to all participants on both test days, and for all test conditions (sitting and during walking). In addition, the gait and cognitive outcome measures are quantified not just for a few seconds, but for durations of minutes.

The absence of a healthy age-matched control group is another limitation. This analysis would provide added information as to differences in DTI due to age effects and disease stage. A study is in progress (paused due to COVID-19) to compare the effects of VMT and VCG on gait function (and *vice versa*) between age-matched able-bodied participants and stage 2 and 3 PD participants.

Finally, the VMT and VCG tasks used in the present study involve both head rotation and information processing, and at this point we cannot rule out any intersegmental mechanical effect of the head rotation as a cause of the gait changes observed between the WO and the DT walk trials. Head rotations required for the VCG tasks were relatively small head rotations of between 20–40 degrees, and of 300–500 ms in duration. These small ramp head rotations about the vertical axis would not likely cause any deviation in position of the body center of mass in ML or AP directions, i.e., passive mechanical disturbance to gait. Duysens et al. (2008) examined center of pressure migration during an open-loop tracking task (up to 30 degrees of visual target motion) while treadmill walking. Three tasks were performed: tracking with eye movements only (head stationary), tracking by rotating the head in synchrony with the moving visual target (open-loop tracking task), and tracking while rotating the trunk in synchrony with the moving visual target. The results demonstrated no significant deviation of the center of pressure migration when participants performed the tracking task with eye or head rotation, whereas, trunk rotations led to a doubling of ML-center of pressure deviation. The mechanical effect of head rotation on gait rhythm pacing and variation will receive further investigation.

## Conclusion

The high to moderate ICC values along with the lack of systematic errors in the measures indicate that this tool has the ability to repeatedly record reliable DTI effects over time. This also proves to be a sensitive tool for tracking disease progression.

A comprehensive analysis of spatial and temporal features of steady state gait has a greater validity to measure gait performance (rhythm, pacing, and stability) as compared to gait speed alone. The use of standardized, interactive computer applications provides a flexible method to produce and evaluate a wide range of executive cognitive activities while performing complex motor behaviors such as walking. Objective evaluation of VMT and VCG computer tasks provide important information about different aspects of information processing.

While the focus of the present work is on assessment of DTI in PD, the GRP platform is directly applicable to other diseases that affect gait and cognition (e.g., cognitive vascular impairment, Alzheimer’s disease, and aging). Future studies will examine the type and levels of DTI that are prognostic of falls and adverse health events. A randomized clinical trial is also planned to examine the feasibility and effectiveness of a computer game-based DT training program for PD patients. Many designed cognitive games, as well as many engaging and inexpensive common and modern computer games can be used with the GRP to challenge a broad range of visuomotor and executive cognitive functions (Mahana et al., 2021).

## Data Availability Statement

The raw data supporting the conclusions of this article will be made available by the authors, without undue reservation.

## Ethics Statement

The studies involving human participants were reviewed and approved by the Human Research Ethics Board at the University of Manitoba. The patients/participants provided their written informed consent to participate in this study.

## Author Contributions

MB, BM, and TS: study concept, design, and acquisition. MB, BM, JK, TK, AK, and TS: analysis, interpretation of data, and preparation of the manuscript. All authors contributed to the article and approved the submitted version.

## Conflict of Interest

The authors declare that the research was conducted in the absence of any commercial or financial relationships that could be construed as a potential conflict of interest.
